# Immune Modulation Through Long‐Term *Lacticaseibacillus rhamnosus* Therapy in Home Mechanically Ventilated Patients

**DOI:** 10.1002/mbo3.70113

**Published:** 2025-11-05

**Authors:** Kamila Szyller, Grazyna Mlynarczyk, Beata Mlynarczyk‐Bonikowska, Janusz Sierdzinski, Joanna Styk, Michal Marusza, Wojciech Marusza

**Affiliations:** ^1^ NZOZ Marusza Clinic Warsaw Poland; ^2^ Department of Medical Microbiology Medical University of Warsaw Warsaw Poland; ^3^ Department of Dermatology Medical University of Warsaw Warsaw Poland; ^4^ Department of Medical Informatics and Telemedicine Medical University of Warsaw Warsaw Poland; ^5^ Department of Dermatology Military Institute of Medicine – National Research Institute Warsaw Poland; ^6^ Faculty of Medicine Lazarski University Warsaw Poland

**Keywords:** cytokines, immunity, immunology, interleukins, *Lacticaseibacillus rhamnosus*, *Lactobacillus rhamnosus*, mechanical ventilation

## Abstract

The beneficial effects of probiotics on the immune system are well established; however, the precise mechanisms underlying their action remain incompletely understood. To date, the impact of probiotics in home mechanically ventilated (HMV) patients has not been investigated. This study evaluated the effects of oral supplementation with *Lacticaseibacillus rhamnosus* GG ATCC 53103 (LGG) on nasal microbiota composition and selected immune parameters in HMV patients. Thirty‐one individuals, following a 3‐month probiotic‐free washout period, received LGG at a dose of 8 × 10^9^ CFU/day for 6 months. Nasal swabs and blood samples were collected at baseline, and after 3 and 6 months, to assess nasal microflora, Th1/Th2 balance, and levels of IL‐2, IL‐4, IL‐5, IL‐10, TNF‐α, and IFN‐γ. A statistically significant increase in IL‐2 was observed at both 3 and 6 months (*p* = 0.0307; *p* = 0.0001, respectively), along with a transient rise in IFN‐γ at 3 months (*p* = 0.0253) and IL‐4 at 6 months (*p* = 0.0297). The IFN‐γ/IL‐10 ratio also increased at 3 months (*p* = 0.0394). No significant changes were detected in the remaining cytokines or nasal bacterial flora. Notably, none of the participants required antibiotic therapy during the intervention period—contrasting with prior seasons, when at least one course was typically necessary. Given the critical role of IL‐2 and IFN‐γ in anti‐infective immunity, their elevation may indicate enhanced resistance to infection, while increased IL‐4 may reflect modulation of inflammation. These findings suggest that LGG supplementation, due to its safety, affordability, and immunomodulatory potential, may be a valuable adjunctive strategy to reduce infection risk and improve outcomes in HMV patients.

## Graphical abstract

Antibiotic resistance crisis drives the search for innovative antibacterial solutions. Probiotics, particularly *Lacticaseibacillus rhamnosus* GG ATCC 53103 (LGG), show promise in preventing bacterial infections and reducing antibiotic use. LGG has well‐documented health benefits and can strengthen the immune system, especially in chronically ill patients. Probiotic supplementation in farm animals stabilizes microbiota and reduces antibiotic dependence. Home mechanical ventilation (HMV) patients face high infection risks, yet LGG's immune benefits in this group remain understudied. This study demonstrates that long‐term LGG therapy enhances immune parameters, particularly by increasing IL‐2 levels. LGG therapy is a cost‐effective and accessible option for improving immunity in HMV patients.

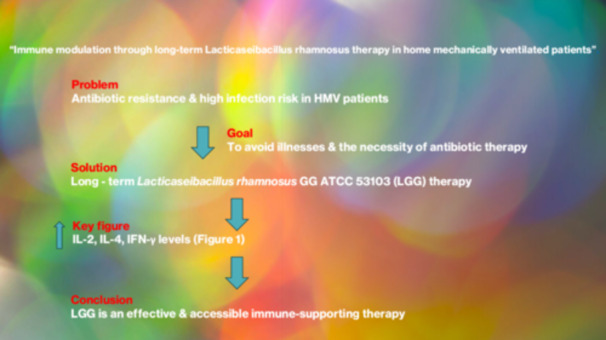

## 1 Introduction

The escalating antibiotic resistance crisis has driven the search for innovative antibacterial solutions. Among these, probiotics have emerged as a promising approach to prevent bacterial infections. However, the immunological mechanisms underlying their therapeutic effects remain enigmatic. For chronically ill patients, it is crucial to explore cost‐effective, innovative treatments.

Numerous studies have examined the use of probiotics, specifically *Lacticaseibacillus rhamnosus* (*L. rhamnosus*) *GG* (LGG) (formerly known as *Lactobacillus rhamnosus*), which colonizes the human digestive system through regular food intake. LGG has been utilized in the treatment of various diseases, including digestive system infections, antibiotic‐associated diarrhea (including *Clostridium difficile*‐induced diarrhea), irritable bowel syndrome, respiratory system infections, allergies, coronary vessel disorders, nonalcohol‐induced hepatic steatosis, hepatitis, cystic fibrosis, and cancers. It has proven to be beneficial in boosting patients' immune systems (Kwok et al. 2022; Capurso 2019; Koscik et al. 2018; Si et al. 2022; Banna et al. 2017). Summarizing 30 years of LGG use, Capurso concluded that LGG promotes a type 1 immune response by reducing the expression of activation and inflammation markers in monocytes, while increasing the production of IL‐10, IL‐12, and TNF‐α in macrophages (Capurso 2019). In recently conducted studies concerning the upper respiratory tract, it has been shown that LGG contributes to the activation of interferon regulatory pathways and the direct inhibition of respiratory viruses (Spacova et al. 2023). *L. rhamnosus* is a well‐studied bacterial species that still holds many mysteries. The mechanism of its beneficial effects on human health is continuously under investigation. In general terms, its impact relies on beneficially modulating the immune system. However, these mentioned studies have overlooked the effects of LGG on the immune system of patients undergoing long‐term probiotic therapy at home, specifically those receiving home mechanical ventilation (HMV). Estimating the number of HMV patients in Europe is challenging, but it is believed that there were approximately 20–100 patients per 100,000 people in 2018 (Winck 2020). In Poland, in 2019, 12,616 patients received HMV treatment, equivalent to approximately 20 per 100,000 individuals (Czajkowska‐Malinowska et al. 2022). HMV treatment was designed to ease the burden on intensive care units by enabling long‐term mechanical ventilation patients to receive care at home (Czajkowska‐Malinowska et al. 2022). These patients are diagnosed with various concurrent medical conditions, and there is a significant risk of death not only from their underlying illnesses but also from complications that may arise during HMV treatment, including bacterial pneumonia (Esteban et al. 2002, 2004). Many patients requiring long‐term mechanical ventilation often present with chronic medical conditions such as neuromuscular disorders (e.g., amyotrophic lateral sclerosis, muscular dystrophy), spinal cord injuries, or severe respiratory conditions (e.g., chronic obstructive pulmonary disease) (Sahetya et al. 2016). These chronic illnesses inherently compromise the immune system, rendering patients more susceptible to infections.

HMV patients may experience immunodeficiency due to a constellation of factors linked to:

− Underlying Medical Conditions: These conditions inherently compromise the immune system, heightening susceptibility to infections.
− Effects of Mechanical Ventilation: The utilization of mechanical ventilation can elicit detrimental effects on respiratory function over time. Barotrauma, volutrauma, and oxygen toxicity represent potential complications associated with mechanical ventilation, further compromising lung function and augmenting susceptibility to infections.
− Prolonged Intubation and Mechanical Ventilation: Both increase the risk of respiratory infections, including ventilator‐associated pneumonia (VAP). The presence of an artificial airway circumvents natural defense mechanisms in the respiratory tract, facilitating pathogen entry into the lungs and subsequent infections.
− Reduced Mobility: Many HMV patients experience limited mobility due to their underlying condition or the necessity for mechanical ventilation. Immobility can contribute to decreased lung function and impaired mucociliary clearance, vital mechanisms for clearing pathogens from the respiratory tract.
− Healthcare Environment and Nutritional Deficiencies: HMV patients frequently necessitate extensive medical interventions and may receive care from multiple healthcare providers, heightening exposure to potential pathogens in healthcare settings. Malnutrition can debilitate the immune system and impair the body's ability to combat infections. Managing these patients necessitates meticulous attention to infection prevention strategies, vigilant monitoring for signs of infection, and the provision of comprehensive supportive care.

It seems that an improvement in the immune system's functioning resulting from LGG supplementation would be a highly anticipated benefit, enhancing the health of the HMV patients. We decided to monitor the overall condition of the patient, some immunological parameters, and the microflora of the nasal cavity, where pathological species dominate in HMV patients. Blood samples and nasal swabs were collected from patients in the morning. We designated Day 0 as the baseline, prior to initiating probiotic treatment. We marked Time 1 as the assessment point after 3 months, followed by Time 2 after 6 months. This approach facilitated the evaluation of changes in immunology and nasal flora within the observed patients. Our selection of the 3‐ and 6‐month timeframes was driven by our extensive clinical experience. We selected the examination of nasal bacterial flora because it was a minimally invasive procedure, swabs were possible to collect from all patients, and results accurately reflected the flora of the patient's nasopharyngeal cavity. Furthermore, considering that some patients had tracheostomies while others did not, we thereby created comparable study conditions. We aimed to determine whether the anticipated positive changes in the immune system's function would impact alterations in the nasal bacterial flora, reducing the number of pathogenic bacterial species.

Considering LGG's documented positive effects on patients' health (Kwok et al. 2022; Capurso 2019; Koscik et al. 2018; Si et al. 2022; Banna et al. 2017) (and in many other publications), along with the Ethical Committee, we decided to conduct this study without a placebo group and considered Day 0 as the baseline day before commencing probiotic treatment. Since all the patients included had not taken any probiotics or yogurts for 3 months prior to the study, they effectively served as their own control group at baseline. With knowledge of the beneficial effects of probiotics or yogurt on human health, we could not ethically deprive a group of severely ill patients of this form of supplementation for a total of 9 months to create a control group. That's why we made the decision as mentioned above.

For this study, we selected a probiotic containing live bacteria *L. rhamnosus* GG ATCC 53103 (1 × 10^9^ CFU in 5 drops) and medium‐chain fatty acids (Acidolac Baby, *L. rhamnosus* GG ATCC 53103 drops by Polpharma). This product is approved in Poland as a medical dietary supplement for infants and children. It is typically used during antibiotic treatment and for a period of 2–3 weeks following antibiotic intake. It is also employed to support the intestinal flora of patients traveling to different climate zones. The product is marketed in a 10 mL bottle. The liquid form ensures safe and accurate administration to HMV patients participating in this study, particularly those with swallowing difficulties who are fed via percutaneous endoscopic gastrostomy (PEG). The product was administered orally in the morning and evening as drops. The dosage was 8 × 10^9^ CFU LGG/day, divided into two equal doses. There are other LGG products available on the Polish market that recommend adult dosages with similar or higher bacteria content. The recommended daily adult dose of LGG in most of these products ranges between 5 and 20 × 10^9^ CFU. A safe recommended adult dose can also reach up to 20 × 10^9^ CFU LGG/day (Wind et al. 2010; Gosselink et al. 2004). Hence, the selected dose for our study followed all known recommendations and was deemed safe for patients.

In summary, the objective of this study was to evaluate the long‐term impact of a probiotic containing LGG ATCC 53103 bacteria on specific parameters of the immune system, such as the levels of selected interleukins (IL) and subpopulations of lymphocytes.

## 2 Materials and Methods

### 2.1 Participants

Thirty‐one patients receiving home mechanical ventilation for more than 8 h a day were recruited. The study was conducted at the Medical University of Warsaw, Poland and NZOZ Marusza Clinic, Warsaw, Poland. The study duration was 6 months, and all participants provided written consent. A total of 29 patients (8 women and 21 men) completed the study and were included in the statistical analysis. Two patients who were ventilated through a PEG tracheostomy did not complete the study due to death from their primary illnesses. Among the 29 patients who completed the study, 23 underwent HMV tracheostomy, with 18 of them having PEG feeding and 5 being fed orally. The remaining 6 patients received noninvasive HMV and were fed orally. On the day of enrollment into the study, patients who had completed it had been receiving HMV treatment for an average of 34.3 months (SD 27.58) and had an average age of 52.4 years (SD 17.36).

### 2.2 Inclusion Criteria

Eligible patients were required to be undergoing HMV treatment and to be in a stable condition, as determined by medical assessments conducted by the internist, neurologist, and anesthesiologist, have no known allergy to the probiotic used, and not have been hospitalized, vaccinated, or treated with antibiotics or probiotics in the 3 months prior to the study. All patients did not consume any yogurts or take any probiotics for a period of 3 months before the study. In this way, they became their own control group. During the study period, patients only consumed the probiotic supplement provided by us, while still abstaining from yogurts and other probiotics not included in the study.

### 2.3 Exclusion Criteria

Patients who developed an allergy to the probiotic during the study, necessitated alterations in chronic drug therapy, required hospitalization, needed antibiotic therapy, or discontinued probiotic use for any reason were excluded from the statistical analysis.

### 2.4 Treatment

Acidolac Baby drops containing *L. rhamnosus* GG ATCC 53103 (LGG), produced by Polpharma, were used in this study. We administered 40 drops (8 × 10^9^ CFU probiotic bacteria) per day, divided into 2 doses of 20 drops in the morning (7 a.m.–9 a.m.) and 20 drops in the evening (6 p.m.–8 p.m.), over a 6‐month period. The product was stored at room temperature and used within 30 days after opening, as per the manufacturer's recommendation. Each patient received a full supply of the medicine free of charge for the 6‐month treatment period. No vaccinations were administered during the study period.

The blood for immunological and hematological tests was collected before the patients received breakfast (between 7 a.m. and 9 a.m.), so the microbiological tests were taken at that time. Blood samples were collected for biochemistry tests, and nasal swabs were taken for microbiological culture at three time points during the study: at the beginning of the study (Time 0), at 3 months (Time 1), and at 6 months (Time 2) while receiving LGG.

### 2.5 Blood Inflammatory Parameters

Blood samples were delivered to the laboratory within a few hours after withdrawal. The choice of cytokines for testing was based on data from previous reports and was restricted by the capabilities of the diagnostic laboratory. The following parameters were tested: erythrocyte sedimentation rate (ESR), C‐reactive protein (CRP), and white blood cell counts. The tests were conducted using Alifax, Alinity ci, Abbott, and SYSMEX XS 1000i devices, respectively.

### 2.6 Cytokine and Lymphocyte Subpopulations

Cytokine concentrations (pg/mL) of the Th1/Th2 profile were measured, including interferon gamma (IFN‐γ), tumor necrosis factor alpha (TNF‐α), and interleukins (IL‐2, IL‐4, IL‐5, IL‐10), along with the ratios: IFN‐γ/IL‐4, TNF‐α/IL‐4, IFN‐γ/IL‐10, and TNF‐α/IL‐10. The commercially available Cytometric Bead Array Th1/Th2 test (BD) was used, enabling simultaneous assessment of these cytokines. The measurements were performed in cell culture supernatants. Lymphocytes isolated from patient blood (collected in lithium heparin, Sarstedt) were separated using a gradient density (Lymphoprep) from peripheral blood and stimulated with a mitogen (PHA, 5 mg/mL, Sigma) for 24 h in a 37°C CO_2_ incubator. The analysis was conducted using the FACSCanto flow cytometer with two lasers (488 and 635 nm, BD) and the FCAP Array 3.0 software. Standard curves were generated using diluted cytokine samples for result reporting.

For the analysis of T cell, B cell, and NK cell subpopulations, the BD Multitest 6‐color TBNK kit with TruCount tubes was used, allowing for the determination of both the percentage and absolute counts of the examined lymphocyte subpopulations. The tests were performed on whole blood (venous blood collected in lithium heparin, Sarstedt) using the methodology recommended by the company (BD). The analysis was conducted using the FACSCanto flow cytometer with two lasers (488 and 635 nm, BD) and the FACSCanto software.

### 2.7 Microbiological Examination

Nasal swabs were collected three times, at the time of blood sample collection from each participant. The samples collected were sent to the Department of Clinical Microbiology, Medical University of Warsaw, within 36 h of collection. The samples were placed in the following media: Columbia agar with 5% sheep blood, mannitol salt agar, MacConkey agar, chocolate agar (Hamophilus chocolate agar), and Schaedler agar (all from BioMaxima SA, Emapol Microbiology Centre, Poland). The cultures were incubated for 24–48 h at 37°C under aerobic, microaerophilic (chocolate agar), and anaerobic (Schaedler agar) conditions. The grown colonies were isolated and evaluated using identification, matrix‐assisted laser desorption/ionization‐time‐of‐flight mass spectroscopy equipped with an IVD Maldi Biotyper system (Bruker Optik GmbH, Ettlingen, Germany), following the manufacturer's instructions.

### 2.8 Statistical Analysis

The SAS 9.4 statistical package was used to statistically evaluate the results obtained. A descriptive analysis of the study results was performed, followed by an assessment of the conformity of the distributions of the studied characteristics to a normal (Gaussian) distribution using the Shapiro–Wilk test. Wilcoxon nonparametric tests, *χ* ^2^ tests, McNemar's test, and Friedman's ANOVA were employed to determine the statistical significance of differences. Spearman's and Cramer's *V* correlation analyses were used to demonstrate the interdependence of the studied characteristics. For all performed analyses, a significance level of *p* < 0.05 was considered.

## 3 Results

The results presented in this study describe the composition of nasal flora in patients receiving chronic mechanical ventilation at home. The nasal flora composition in almost all the individuals studied differed significantly from that of healthy individuals and more closely resembled the flora of hospitalized patients. In 41% of the patients, no coagulase‐negative *Staphylococci* were obtained, which is typical for this region, in any culture. *Staphylococcus aureus* was cultured in 12 patients (41%). Most patients presented massive colonies of Gram‐negative bacteria, predominantly *Pseudomonas aeruginosa* (*P. aeruginosa*; 18 patients, 62%), and Enterobacteriales, particularly *Klebsiella pneumoniae* (*K. pneumoniae*) and *Klebsiella oxytoca* (10 patients, 34%), as well as various species of *Proteus*, mainly *Proteus mirabilis* (8 patients, 28%). While coagulase‐negative *Staphylococci* were detected in patients based on a single culture, the same Gram‐negative species were present in all three or at least two swabs taken from the same patient. The results are presented in Table 1.

**Table 1:** Isolates of bacterial species present in cultures taken from the nasal cavity of 29 patients: before probiotic administration (Time 0), at 3 months (Time 1), and at 6 months (Time 2).

Species/genus	Total number of isolates			Total number of patients colonized^a^	Patients stably colonized^b^
	Time 0	Time 1 (3 months)	Time 2 (6 months)		
*Gram‐negative*					
Nonfermenters					
*Pseudomonas aeruginosa*	12	15	13	18	14
*Pseudomonas* spp.	1	0	1	2	0
*Stenotrophomonas maltophilia*	0	1	2	3	0
*Acinetobacter* spp.	5	6	1	8	3
*Kerstersia gyiorum*	0	0	1	1	0
*Elizabethkingia meningoseptica*	0	1	0	1	0
*Moraxella catarrhalis*	1	0	0	1	0
Enterobacteriales					
*Klebsiella pneumoniae*	6	9	8	9	8
*Klebsiella oxytoca*	2	1	1	2	1
*Enterobacter* spp.	2	2	1	2	2
*Serratia marcescens*	3	5	6	6	5
*Proteus* spp.	6	6	7	8	7
*Morganella morganii*	3	3	5	5	5
*Providencia stuartii*	1	2	3	3	2
*Citrobacter* spp.	2	1	0	2	1
*Escherichia coli*	0	1	1	1	1
*Pantoea agglomerans*	0	0	1	1	0
*Gram‐positive*					
*Staphylococcus aureus*	8	5	9	12	5
Coagulase‐negative *Staphylococci*	9	5	7	17	3
*Rothia mucilaginosa*	1	0	0	1	0
*Corynebacterium* spp.	6	3	4	10	3
*Streptococcus pneumoniae*	1	0	1	1	1
Oral *Streptococci*	0	2	0	2	0
*Aerococcus viridans*	0	0	1	1	0
*Propionibacterium acnes*	1	0	0	1	0

a Detected at least once in each patient.

b Detected at least twice in the same patient.

No statistically significant changes were observed in the composition of nasal microflora during probiotic therapy. A significant stability in colonization by certain bacterial species, especially *P. aeruginosa*, was noted, with this microorganism cultured from at least 14 patients on two or more occasions, and *K. pneumoniae* cultured from 8 patients. The decrease in the number of *Acinetobacter* spp. cultures in the third examination is difficult to explain.

Overall, our immunological and hematological findings indicated higher than normal levels of ESR, CRP, TNF‐α, IL‐2, and ratios for TNF/IL‐4 and TNF/IL‐10, and lower than normal levels of IL‐10 and the IFN/IL‐4 ratio, with the IFN/IL‐10 ratio being lower only at Time 0 (Table S1).

**Figure 1:**
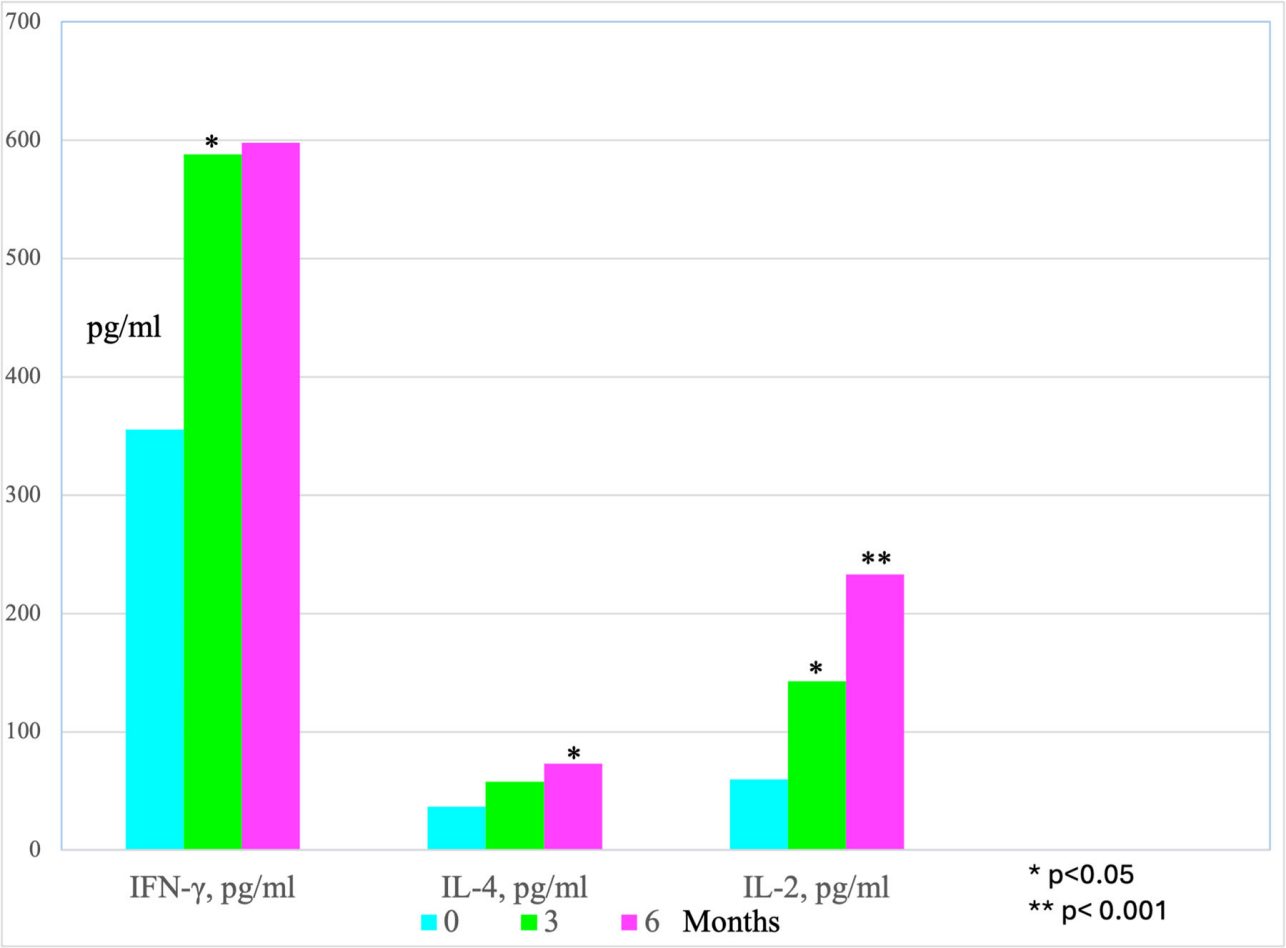
Statistically significant changes in immunological parameters compared to Time 0 values, average values.

**Figure 2:**
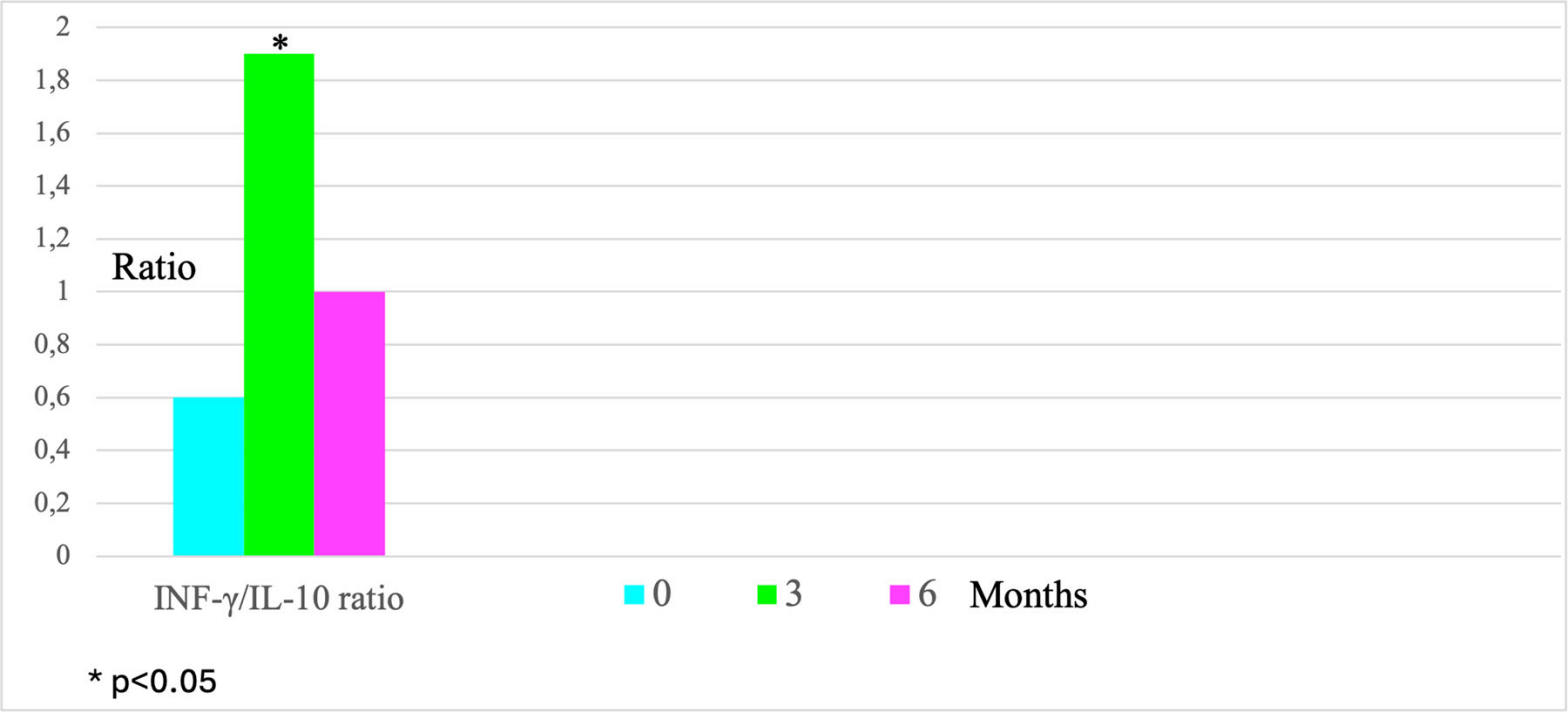
Statistically significant changes in immunological parameters compared to Time 0 values, average values.

Significant changes were observed after 3 months of probiotic intake compared to Time 0. There was an increase in IFN‐γ levels from 355.4 to 588.0 pg/mL (*p* = 0.0253), which, however, remained within the normal range of 290–1050 pg/mL (Figure 1). IL‐2 levels increased from 59.6 to 142.8 pg/mL (*p* = 0.0307), exceeding the normal range of 20.0–45.0 pg/mL (Figure 1). The IFN/IL‐10 ratio also increased from 0.6 to 1.9 (*p* = 0.0394), surpassing the normal range of 1.1–2.8 (Figure 2). These changes indicate the induction of pro‐inflammatory cytokine stimulation. Notably, the significant increase in IL‐2 levels, well beyond what is observed in normal individuals, suggests a significant role of IL‐2 in the immunological response (Table S1).

After 6 months of probiotic intake, IL‐4 levels increased from 36.9 to 73.0 pg/mL (*p* = 0.0297), remaining within the normal range of 20.0–120.0 pg/mL (Figure 1). However, IL‐2 levels increased from 59.6 to 233.2 pg/mL (*p* = 0.0001), significantly surpassing the normal range of 20–45 pg/mL (Figure 1). The substantial increase in IL‐2 levels after 6 months of probiotic intake likely indicates further immune system stimulation. The smaller increase in IL‐4 levels remained within the normal range and was likely secondary to the rise in IL‐2 (Figure 1).

None of the patients who completed the study required: antibiotic therapy (whereas previously, in similar period and time of the year, they typically required at least one course of antibiotic therapy), hospitalization, or temporary discontinuation of Acidolac Baby drops. Two patients died at home due to the progression of their primary illnesses after 3 months of the study; they were not included in the analysis. The 29 patients who completed the study remained in stable medical condition throughout the probiotic intake period, which can be considered a successful outcome. The immunological and hematological findings are presented in Table S1. Statistically significant changes in immunological parameters are presented in Figures 1 and 2.

## 4 Discussion

Our work constitutes a scientific investigation rather than a clinical trial, conducted by a cohort of practicing physicians to validate specific hypotheses outlined in the study's introduction. Consequently, it did not strictly adhere to established clinical trial protocols. The relatively small size of our study group stems from several factors. First, patients undergoing home mechanical ventilation (HMV) represent a rare subgroup within the general population, and their considerable geographical dispersion further limited recruitment. Additionally, the number of patients included reflects the limited pool of individuals receiving HMV under our direct care during the study period, combined with the necessity of obtaining informed consent and ensuring adherence to study protocols. The study population was also ethnically homogeneous, consisting of patients from a single geographical region (Silesian Voivodeship, Poland), which, while limiting generalizability, helped control for potential confounding factors related to ethnic and regional diversity.

Each patient remained in their home environment, ensuring consistent, stable care conditions. We aimed to test a specific hypothesis, which we achieved by conducting what we believe to be a rigorous study, documented with appropriate statistical tests tailored to the number and type of patients involved.

Participation in the study necessitated that HMV patients meet specific criteria: they had to maintain a stable medical condition, possess no known allergy to the probiotic utilized, and refrain from hospitalization, vaccination, or antibiotic/probiotic treatment for 3 months preceding the study. Additionally, patients abstained from consuming yogurt or probiotics for 3 months prior to the study. Throughout the study period, patients exclusively ingested the provided probiotic supplement while refraining from yogurt and other nonstudy probiotics, as detailed in Section 2. The fulfillment of these criteria, alongside the study's sample size and tailored statistical analyses, mitigated randomness and ensured the reliability of study results, which could be compared against baseline data obtained at the study's inception. Thus, patients effectively served as their own control group.

Gram‐positive bacillus LGG, which colonizes the human digestive system, is one of the most widely tested probiotic strains and has been known for many years (Capurso 2019). With the increase in drug resistance and extended lifespan of chronically ill patients due to advanced technologies and treatment, strengthening the immune system using a noninvasive and safe approach has become crucial for therapy. Patients undergoing chronic mechanical ventilation face heightened mortality risks (Esteban et al. 2002, 2004; Sahetya et al. 2016). Those receiving mechanical ventilation (MV) for over 48 h have a tenfold higher probability of developing pneumonia compared to nonventilated patients in intensive care units (American Thoracic Society 2005; Jorens 2016). VAP is typically caused by bacteria that colonize the respiratory system of such patients. MV leads to ventilator‐induced lung inflammation, which is dependent on tidal volume (VT). This inflammation is characterized by the activation of pro‐inflammatory Th1 cytokines, including TNF‐α, IL‐1β, IFN‐γ, and IL‐6, as well as the infiltration of innate cells such as neutrophils and macrophages into the lung (Parker et al. 1993; Gajic et al. 2004; Tremblay et al. 1997; Neto et al. 2012; Bielen et al. 2018; Wienhold et al. 2018). Even with a “lung protective” ventilation strategy, rats still exhibit increased Th1 cytokines and an anti‐inflammatory Th2 response with elevated IL‐4 secretion (Bielen et al. 2018). Notably, blocking IL‐4 signaling normalized lung bacterial burden in VAP‐induced animals (Bielen et al. 2018). Therefore, while Th2 cytokines may contribute to transient immunosuppression in ventilated lungs, promoting bacterial and fungal growth, the downstream mechanisms remain unknown (De Winter et al. 2019).

Maintaining an appropriate balance between pro‐inflammatory and anti‐inflammatory cytokines is crucial, as an increase in Th2 cytokines for patients undergoing MV treatment can be harmful. LGG, as a safe and harmless cytokine modulator, has been described in previous studies as a positive influencer of inflammatory processes (Zamojska et al. 2021; Han et al. 2021; Zhang et al. 2018; H. Wang et al. 2016; B. Wang et al. 2020; Khailova et al. 2014; Ludwig et al. 2018; Sindhu et al. 2014). Capurso, summarizing 30 years of LGG use, concluded that LGG promotes a type 1 immune response by reducing the expression of activation and inflammation markers in monocytes, while increasing the production of IL‐10, IL‐12, and TNF‐α in macrophages (Capurso 2019).

Our study detected a significant increase in IL‐2 production associated with the promotion of Th1‐type responses. The extensive evidence supporting the use of LGG for human health suggests that introducing a probiotic therapy containing LGG to patients with chronic diseases can improve their immune system, reduce the frequency of infections, and minimize the need for antibiotic treatment. This is particularly crucial in the context of increasing antibiotic resistance.

While most medical literature on LGG and the immune system focuses on animals, successful supplementation of lactic acid bacteria‐containing probiotics in farm animals has shown stabilization of the intestinal microbiota and protection against pathogenic bacteria, leading to reduced antibiotic use and improved growth and food intake (Zamojska et al. 2021). Studies on mouse models have shown that LGG supplementation enhances the immune response by activating innate immunity, suppressing systemic inflammation, and modulating the gut microbiota (Han et al. 2021; Zhang et al. 2018; H. Wang et al. 2016; B. Wang et al. 2020). LGG has also demonstrated a significant effect on the modulation of the inflammatory response and cytokine release in mouse models (Khailova et al. 2014). The influence of LGG on the activation of innate immunostimulation and cytokine responses has been confirmed in various studies (H. Wang et al. 2016; Ludwig et al. 2018; Sindhu et al. 2014). While these studies primarily focus on the digestive or respiratory systems, our study aimed to investigate lymphocyte populations, cytokines, and blood biochemistry in patients receiving probiotic therapy.

In our study, we observed higher levels of inflammatory indicators and elevated ESR, CRP, TNF‐α, and IL‐2 levels, as well as TNF/IL‐4 and TNF/IL‐10 ratios, in the blood serum of patients. After 3 months of probiotic usage, we observed a significant increase in IL‐2 levels, which further increased after 6 months. The question arises, on the one hand, about the mechanism in which *L. rhamnosus* influences increased levels of IL‐2 and IFN gamma and, on the other hand, how this is related to improved immunity to infection in the patients studied. As previously studied, bacteria in probiotics including *L. rhamnosus* probably act through TLR9 to affect dendritic cells, which in turn modify the function and response of T lymphocytes. This modification, as it seems, may be of different nature in different study groups and undoubtedly depends on many factors including the age of the patients, the state of their immune system, comorbidities, antigenic stimulation, etc. The results on the effect of *L. rhamnosus* on the concentration of IL‐2 also vary in different groups, and so, for example, in patients with inflammatory bowel diseases under the influence of probiotics, the concentration of IL‐2 decreases while in patients with systemic lupus an increase in the concentration of this cytokine is observed. In both cases, a beneficial effect on the clinical course of these diseases was observed. In the first case, it was explained by a decrease in inflammation and an improvement in intestinal tightness, while in the second case, it was explained by the stimulatory effect of IL‐2 on Treg lymphocytes. In a group of healthy athletes taking probiotics containing multiple bacterial strains including *L. rhamnosus*, despite the effect on the concentration of some other cytokines, for example, an increase in IFN gamma concentration, there was no effect on IL‐2. The effect of IL‐2 itself depending on the concentration of the cytokine, antigenic stimulation, the presence of other cytokines, target immune cells and other factors present in the microenvironment can vary from immunoregulatory (low concentrations in the above‐mentioned SLE patients), immunostimulatory effect of higher concentrations in anti‐infective or antitumor responses to the harmful effect of extremely high concentrations produced under the influence of bacterial superantigens in toxic shock syndrome. To our knowledge, the effect of probiotics on the immune system has not yet been studied in a group of in HMV patients. The protective effect against infections in our patients, whose IL‐2 concentrations were relatively high, may be related to the stimulation of the production of stimulated growth and differentiation of T lymphocytes, the stimulation of their production of IFN gamma, and the effect of IFN gamma and IL‐2 on the one hand on cell‐type responses such as cytotoxic T lymphocytes or NK cells on the other hand on the production of IgG and IgM antibodies by B lymphocytes (You et al. 2014; Braat et al. 2004; Alaei et al. 2023; Jounai et al. 2012; Guo et al. 2022; Arenas‐Ramirez et al. 2015). IL‐2 and IFN gamma act synergistically and can also stimulate each other's production by immune system cells through positive feedback. They act via the JAK1/3–STAT5 and JAK1/2–STAT1 signaling pathways. This leads to the differentiation and activation of Th1 lymphocytes, which play an important role in the anti‐infective response. Both cytokines play a key role in the response to viral, bacterial, and fungal infections, as well as in fighting cancer. IL‐2 has been approved for cancer immunotherapy and is being investigated for chronic viral infections. It has been shown to have antitumor responses against lung tumor nodules, and its production has been associated with acupuncture point stimulation in patients with lung cancer. IL‐2 has multiple functions and is essential for immune responses, orchestrating cellular interactions, and shaping the nature and magnitude of immune responses. Manipulating IL‐2 levels in patients is a focus of immunotherapeutic approaches, ranging from inhibition of IL‐2 for immunosuppression to its application as a vaccine adjuvant in cancer therapies (Abbas 2020; Walser et al. 2018; Bendickova and Fric 2020). Thus, the increase in IL‐2 levels observed in patients undergoing LGG probiotic treatment should be considered a positive effect of this therapy, suggesting both enhanced immune response against infectious agents and potential anti‐inflammatory effects. The increase in IFN gamma concentration also has a beneficial effect, primarily by stimulating the response to infections.

Additionally, there was an increase in IL‐4 levels within the normal range. The effect of LGG on IL‐4 production depends on the conditions. In patients with atopic allergies and in animal models of allergies, the production of this cytokine has been inhibited. On the other hand, in a study of isolated bovine splenocytes, stimulation with LGG not only increased IL‐2 transcription (*p* < 0.05) ~30‐fold, but also increased IL‐4, IL‐10, and IL‐17 transcription (Brasil et al. 2024) It has been shown that the binding of IL‐2 among others to IL2R activates JAK1/3 signaling, leading to the phosphorylation of signal transducer and transcription 5 (STAT5), which can consequently result in increased expression of IFNγ but also IL‐4 (Damoiseaux 2020). The role of IL‐4 is more difficult to explain. It is a cytokine that acts as an antagonist to IL‐2 and IFN gamma, primarily via the IL‐4R–JAK1/3–STAT6 signaling pathway. Perhaps in the patients we studied, it played a role in inhibiting the excessive inflammatory response caused by IL‐2 and IFN gamma. Conversely, elevated concentrations of the latter two cytokines may have shielded the studied patients from the reduction in anti‐infective immunity triggered by elevated IL‐4 levels. All patients who completed the study remained in a stable medical condition throughout the entire observation period. None of the patients receiving probiotic therapy required antibiotic treatment, whereas previously, in a similar period and time of the year, they typically required at least one course of antibiotic therapy. Microbiological examinations revealed the presence of potential pathogens, likely associated with the multiple hospitalizations that patients had previously experienced. It is known that prolonged hospitalization causes a shift in the normal flora on the mucous membranes of the respiratory system toward Gram‐negative bacteria (Esposito et al. 2003). In our study, the presence of Gram‐negative bacilli was observed in the nasal cavity swabs of almost all the patients. Many species are the most common etiological agents of VAP. However, the therapy did not influence the presence or composition of the nasal flora, suggesting that the absence of infections and morbidity was likely due to enhanced immunity rather than the elimination of potential pathogens. Despite our understanding that the nasal and oropharyngeal bacterial flora remains unaffected by oral probiotic administration, we opted to monitor it meticulously to ensure clarity and dispel any doubts pertaining to the outcomes of immunological evaluations. Furthermore, we endeavored to explore whether prolonged LGG therapy could induce alterations in nasal bacterial flora through its impact on patient immunology, potentially mitigating the presence of pathogenic bacterial species. The lack of noticeable changes in the nasal and oropharyngeal bacterial flora among patients confirms that the observed improvement in immunity is solely due to the alterations noted in immunological parameters.

## 5 Conclusions

Long‐term LGG therapy for HMV patients is associated with a significant increase in IL‐2 and IFN gamma production, leading to improved immunity and a reduced need for antibiotic therapy. This is particularly crucial in the context of increasing antibiotic resistance. Therefore, LGG therapy has the potential to influence clinical practice and shape public policy, making it a recommended cost‐effective and accessible approach for HMV patients. However, it is important to note that further research with a larger number of patients is required to obtain more conclusive data on the positive impact of higher IL‐2 levels. Nevertheless, our study demonstrated highly positive outcomes with LGG therapy in the evaluated patients, which can likely be attributed to the significant increase in Interleukin‐2 and IFN gamma. The fact that IL‐2 treatment has been approved by the United States Food and Drug Administration for cancer immunotherapy and is currently being tested in clinical trials for the treatment of chronic viral infections further supports our recommendation of LGG therapy for patients undergoing HMV treatment.

In conclusion, we strongly recommend LGG therapy as a beneficial supportive treatment and prophylaxis for HMV patients. It not only reduces the risk of infections but also improves overall health. As a cost‐effective and easily accessible option, LGG therapy could play a significant role in enhancing the well‐being of HMV patients.

## Author Contributions

**Kamila Szyller:** conceptualization, investigation, funding acquisition, writing – original draft, methodology, validation, visualization, writing – review and editing, formal analysis, project administration, data curation, resources. **Grazyna Mlynarczyk:** conceptualization, investigation, writing – original draft, methodology, validation, visualization, writing – review and editing, formal analysis, supervision. **Beata Mlynarczyk‐Bonikowska:** conceptualization, investigation, writing – original draft, methodology, validation, visualization, writing – review and editing, formal analysis, project administration, data curation. **Janusz Sierdzinski:** conceptualization, investigation, writing – original draft, methodology, validation, visualization, writing – review and editing, software, formal analysis, data curation. **Joanna Styk:** investigation, writing – original draft, methodology, validation, visualization, writing – review and editing, formal analysis, data curation. **Michal Marusza:** data curation, methodology, investigation, formal analysis, validation, visualization, writing – review and editing, writing – original draft. **Wojciech Marusza:** conceptualization, investigation, funding acquisition, writing – original draft, methodology, validation, visualization, writing – review and editing, formal analysis, project administration, resources, supervision, data curation. All authors read and approved the final manuscript. All authors contributed to this study, design of the study, collection, data analysis, interpretation of data, writing manuscript, and revising the paper.

## Ethics Statement

The study was approved by the Ethics Committee at the Regional Medical Chamber in Warsaw (Number KB1407/22; Date of Registration 29.09.2022) and was conducted in accordance with the Declaration of Helsinki.

## Consent

Informed consent was obtained from all subjects involved in the study. All participants provided their written informed consent.

## Conflicts of Interest

The authors declare no conflicts of interest.

## Supporting information


**Supplemental Table:** Immunological and Hematological results in SI.

## Data Availability

All available data are presented in the tables in this manuscript.
