# Supplementary material for: Immune Modulation Through Long‐Term Lacticaseibacillus rhamnosus Therapy in Home Mechanically Ventilated Patients
Source: Microbiologyopen. 2025 Nov 5;14(6):e70113. doi: 10.1002/mbo3.70113 (PMC12587047; doi:10.1002/mbo3.70113)
Supplement: Supplementary file 1 — Supplemental Table: Immunological and Hematological results in SI. [file MBO3-14-e70113-s001.docx]

**Supplemental Table:** Immunological and Hematological results in SI

| **Laboratory test** | **Statistical**  **significance**  **p value 0 to 1** | **Statistical significance**  **p value 0 to 2** | **Statistical significance**  **p value 1 to 2** | **NORM** | **Average**  **Time 0** | **Average**  **Time 1**  **(3 months)** | **Average**  **Time 2**  **(6 months)** | **SD**  **Time 0** | **SD**  **Time 1** | **SD**  **Time 2** |
| --- | --- | --- | --- | --- | --- | --- | --- | --- | --- | --- |
| ESR, mm/h |  |  |  | 0-15 | 31.1 | 29.9 | 30.9 | 19.47 | 16.84 | 18.03 |
| CRP, mg/l |  |  |  | 0-5 | 16.1 | 12.4 | 14.6 | 28.36 | 15.46 | 26.17 |
| WBC, x10^3^/ul |  |  |  | 3.8-10.0 | 8.3 | 8.0 | 7.8 | 3.62 | 3.29 | 3.23 |
| T CD3, % |  |  |  | 59.7-82.0 | 72.5 | 71.1 | 71.0 | 16.86 | 17.25 | 16.91 |
| T CD3, μl |  |  |  | 900.0-2600.0 | 1307.1 | 1264.9 | 1212.8 | 637.94 | 588.43 | 560.76 |
| B CD19, % |  |  |  | 7.2-22.5 | 9.8 | 8.7 | 8.9 | 6.42 | 5.35 | 5.72 |
| B CD19, μl |  |  |  | 100.0-600.0 | 172.0 | 154.2 | 145.8 | 144.08 | 109.56 | 109.02 |
| Helper CD3/CD4, % |  |  |  | 30.4-51.2 | 43.9 | 43.2 | 43.5 | 12.10 | 12.58 | 11.79 |
| Helper CD3/CD4, μl |  |  |  | 500.0-1600.0 | 772.2 | 761.2 | 733.3 | 341.45 | 365.97 | 326.22 |
| Suppressor CD3/CD4, % |  |  |  | 19.0-38.9 | 26.8 | 26.7 | 26.4 | 13.24 | 12.56 | 11.24 |
| Suppressor CD3/CD4, μl |  |  |  | 300.0-1200.0 | 501.6 | 475.1 | 454.8 | 348.44 | 296.40 | 279.61 |
| CD4/CD8, ratio |  |  |  | 0.8-2.5 | 2.6 | 1.9 | 1.9 | 3.38 | 1.32 | 1.21 |
| NK cells, % |  |  |  | 7.3-24.0 | 13.4 | 16.1 | 16.3 | 8.52 | 10.20 | 10.25 |
| NK cells, μl |  |  |  | 100.0-500.0 | 233.2 | 269.6 | 271.4 | 141.03 | 171.39 | 200.26 |
| **IFN-γ, pg/ml** | **p<0,05** |  |  | 290.0-1050.0 | **355.4** | **588.0** | 598.1 | 347.26 | 450.99 | 670.59 |
| TNF-α, pg/ml |  |  |  | 320.0-1380.0 | 1516.2 | 2193.9 | 2100.1 | 1396.85 | 1553.42 | 1482.19 |
| IL-10, pg/ml |  |  |  | 1530.0-3830.0 | 703.1 | 777.6 | 884.5 | 471.02 | 770.46 | 827.74 |
| **IL-4, pg/ml** |  | **p<0,05** |  | 20.0-120.0 | **36.9** | 57.7 | **73.0** | 33.98 | 65.34 | 83.17 |
| IL-5, pg/ml |  |  |  | 20.0-95.0 | 23.7 | 29.5 | 31.8 | 15.91 | 24.48 | 30.93 |
| **IL-2, pg/ml** | **p<0,05** | **p<0,001** |  | 20.0-45.0 | **59.6** | **142.8** | **233.2** | 67.84 | 196.00 | 223.28 |
| INF-γ/IL-4 ratio |  |  |  | 47.3-135.8 | 11.1 | 15.4 | 12.3 | 9.54 | 13.70 | 11.18 |
| TNF- α/IL-4 ratio |  |  |  | 3.2-31.2 | 49.4 | 59.3 | 53.4 | 52.34 | 52.02 | 47.61 |
| **INF-γ/IL-10 ratio** | **p<0,05** |  |  | 1.1-2.8 | **0.6** | **1.9** | 1.0 | 0.60 | 3.23 | 1.45 |
| TNF- α/IL-10 ratio |  |  |  | 0.2-0.5 | 2.8 | 7.4 | 4.2 | 3.00 | 12.53 | 4.78 |
| **Laboratory test** | **Statistical**  **significance**  **p value 0 to 1** | **Statistical significance**  **p value 0 to 2** | **Statistical significance**  **p value 1 to 2** | **NORM** | **Average**  **Time 0** | **Average**  **Time 1**  **(3 months)** | **Average**  **Time 2**  **(6 months)** | **SD**  **Time 0** | **SD**  **Time 1** | **SD**  **Time 2** |

p not written - statistically insignificant; the values that underwent statistically significant changes have been written in bold font.
